# Supplementary material for: Transcription elongation factor AFF2/FMR2 regulates expression of expanded GGGGCC repeat-containing C9ORF72 allele in ALS/FTD
Source: Nat Commun. 2019 Nov 29;10:5466. doi: 10.1038/s41467-019-13477-8 (PMC6884579; doi:10.1038/s41467-019-13477-8)
Supplement: Supplementary file 1 — Supplementary Information [file 41467_2019_13477_MOESM1_ESM.pdf]

Supplementary Information

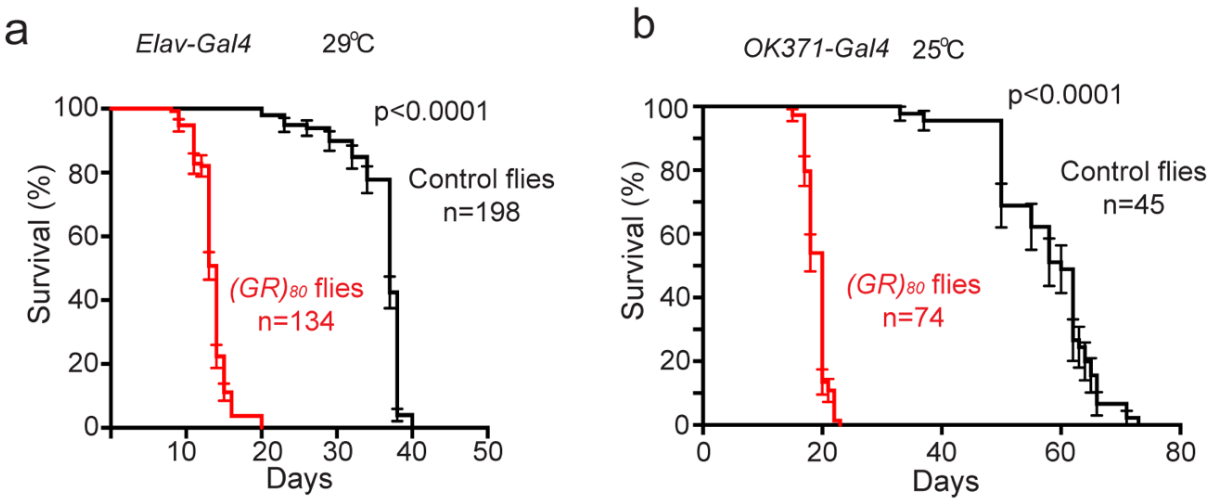

Supplementary Figure 1. Survival analysis of pan-neuronal $(GR)_{80}$ expression at 29°C (**a**) or motor neuron-specific $(GR)_{80}$ expression at 25°C (**b**). The genotypes are: *ELAV-Gal4-Gal80^{ts}; UAS-(CONT-GR)$_{80}$/w^{1118}* vs. *ELAV-Gal4-Gal80^{ts}::UAS-(GR)$_{80}$/w^{1118}* at 29°C; *OK371-Gal4-Gal80^{ts}; UAS-(CONT-GR)$_{80}$/w^{1118}* vs. *OK371-Gal4-Gal80^{ts}::UAS-(GR)$_{80}$/w^{1118}* at 25°C. The number of flies for each genotype is indicated. $p < 0.0001$ by Log-rank test. Source data are provided as a Source Data file.

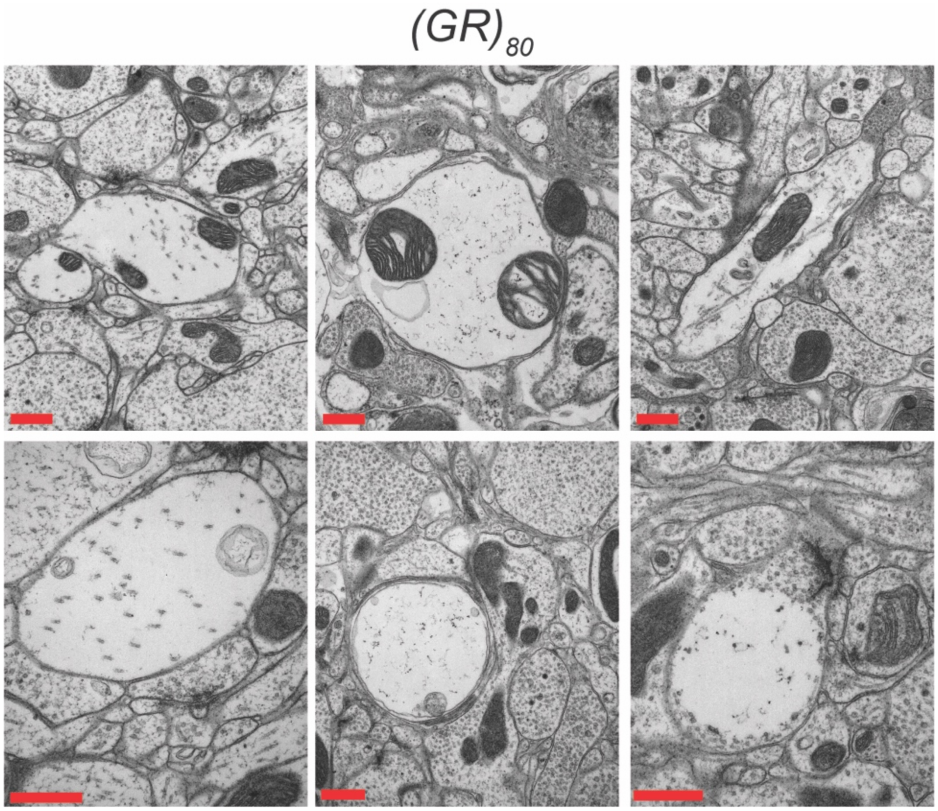

Supplementary Figure 2. Electron microscopic images of the ventral ganglion neuropil in *(GR)*$_{80}$ flies showing degenerating axons. Scale bars: 0.5 µm.

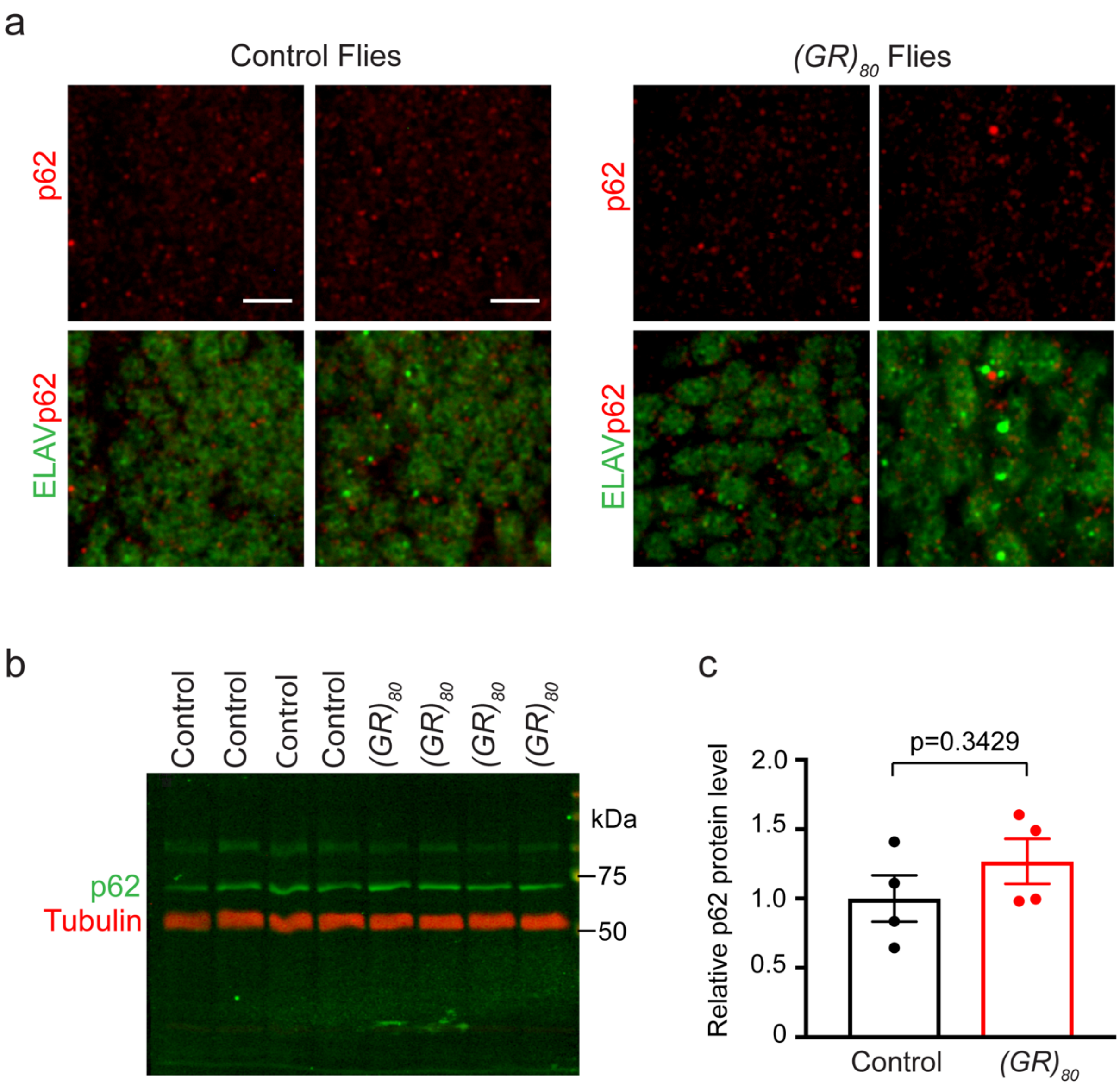

Supplementary Figure 3. $(GR)_{80}$ expression does not induce p62 pathology in the *Drosophila* brain. **a** Immunostaining of three weeks old control and *$(GR)_{80}$* flies with anti-ELAV (green) and anti-p62 (red) antibodies. P62 did not accumulate in $(GR)_{80}$ expressing neurons. Scale bar: 5 µm. **b** Western blot analysis of 3-week-old control and *$(GR)_{80}$* fly brains. Each lane represents an independent cross. **c** p62 protein level (n = 4 samples from independent crosses). Values are mean ± s.e.m. p = 0.3429 (Mann-Whitney test). Source data are provided as a Source Data file.

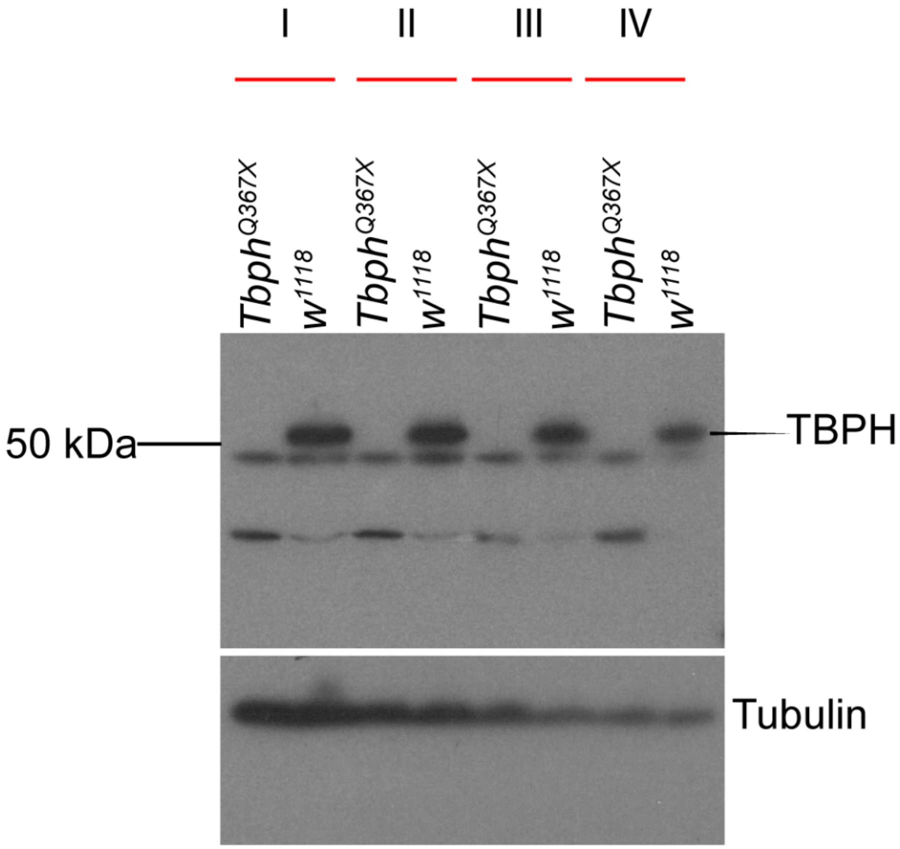

Supplementary Figure 4. Anti-TBPH/dTDP-43 antibody is specific for *Drosophila* TBPH protein. Western blot analysis of w^{1118} and $TBPH^{Q367X}$ larval lysates showing absence of the 55-kDa TBPH band in $TBPH^{Q367X}$ mutants. Source data are provided as a Source Data file.

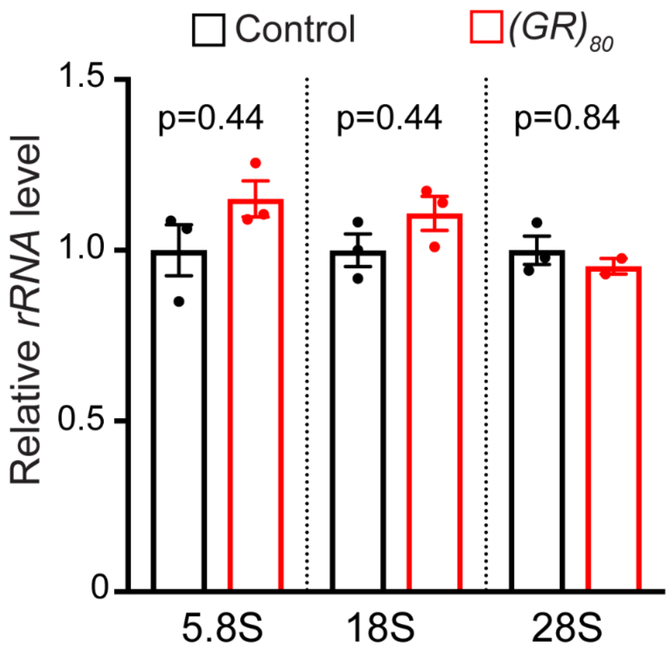

Supplementary Figure 5. $(GR)_{80}$ expression does not induce nucleolar stress in *Drosophila*. QPCR analysis of 5.8S, 18S and 28S rRNA levels in the brain of 3-week-old control and *$(GR)_{80}$* flies. Each data point is from an independent cross (n = 3 independent crosses). Values are mean ± s.e.m. (multiple *t* test, corrected for multiple comparisons using Holm-Sidak method)**.** Source data are provided as a Source Data file.

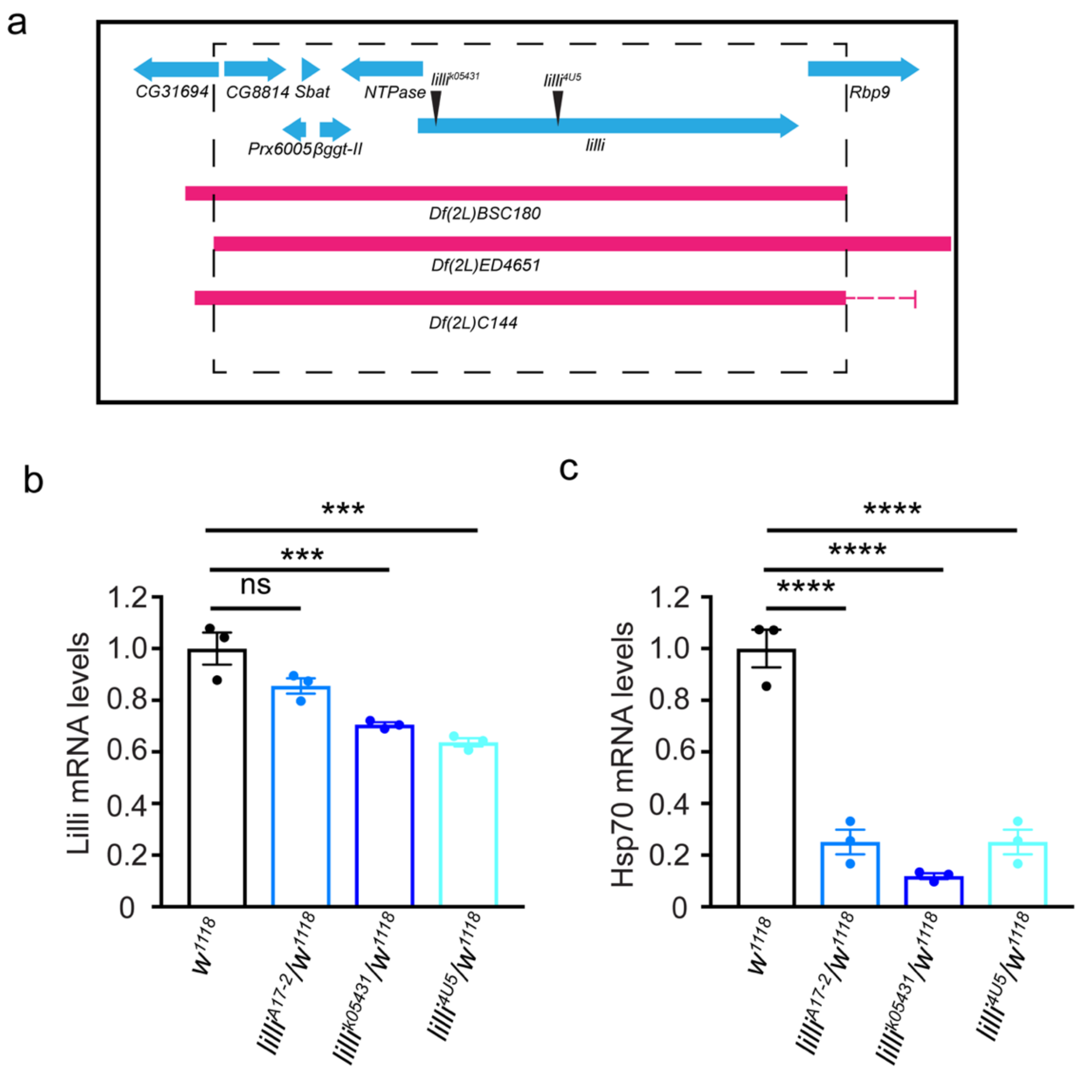

Supplementary Figure 6. a Schematic of the locus covered by *Df(2L)C144*, *Df(2)ED4651*, and *Df(2)BSC180* (adapted from Flybase). Dashed box indicates the region common to all three deficiency lines. The location of the *lilli* alleles were indicated with the triangle. b QPCR analysis of *lilli* mRNA levels in w^{1118} and heterozygous *lilli* alleles; $lilli^{A17\text{-}2}$, lilli^{k05431} and lilli^{4U5}. Values are mean ± s.e.m. ***$p < 0.001$ (one-way ANOVA, Dunnett's multiple comparison test). c QPCR analysis of *hsp70* mRNA levels after 30' heat shock treatment. Values are mean ± s.e.m. ****$p <$ 0.001 (one-way ANOVA, Dunnett's multiple comparison test). Each data point is an independent cross in b and c (n = 3 independent crosses). Source data are provided as a Source Data file.

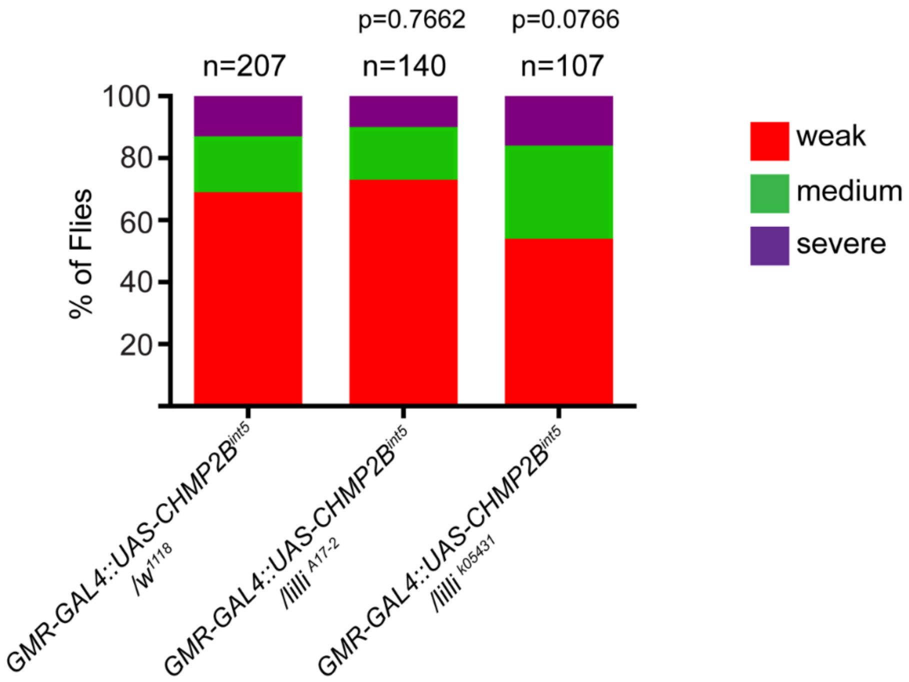

Supplementary Figure 7. *lilli* alleles don't modify *CHMP2B*^{int5} toxicity. Expression of *UAS-CHMP2B*^{int5} in fly eye generates rough eye phenotype with varying severity [1]. The chart shows the quantification of rough eye phenotype severity in flies with wild type and one copy mutant *lilli* alleles (Fisher's exact test). Source data are provided as a Source Data file.

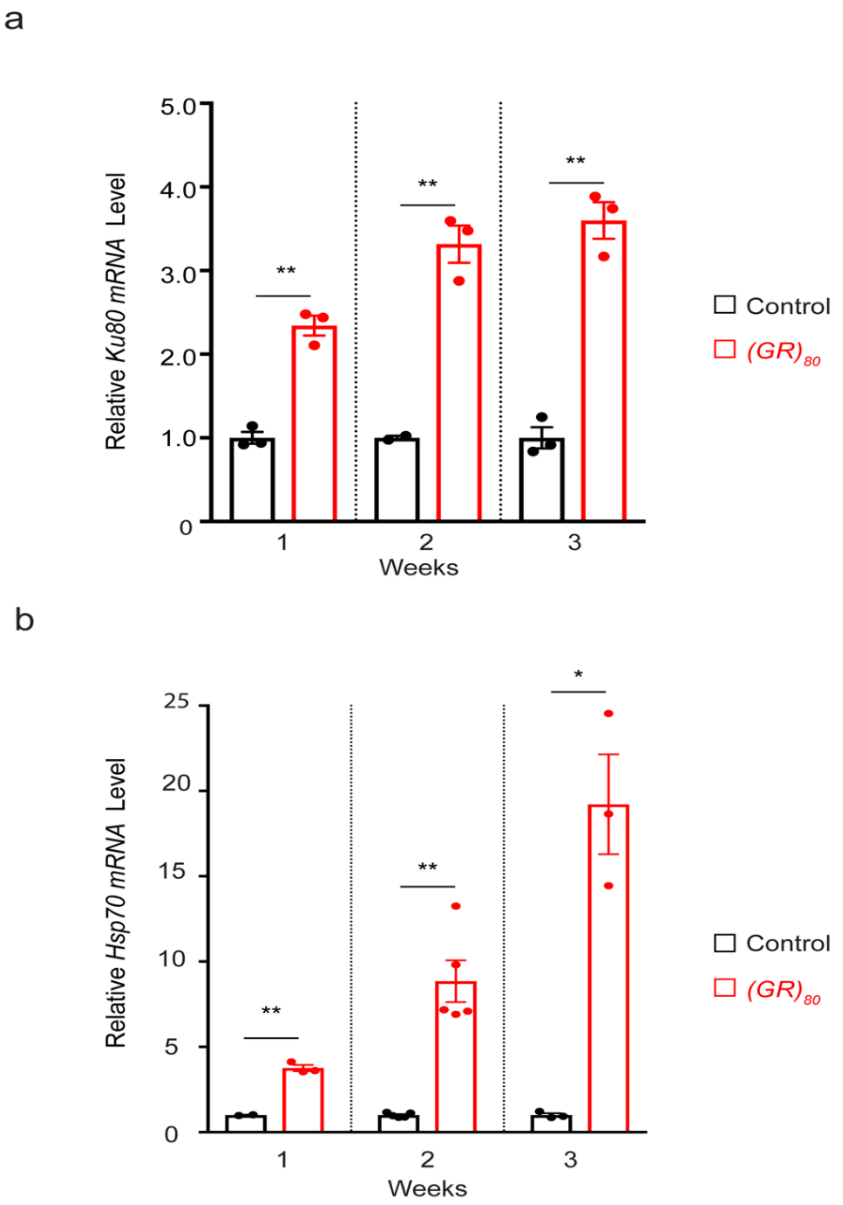

Supplementary Figure 8. $(GR)_{80}$ expression induces upregulation of *Ku80* and *Hsp70***.** QPCR analysis of *Ku80* (**a**) and *Hsp70* (**b**) mRNA levels in 1-, 2- and 3-week-old fly heads. Each data point is from an independent genetic cross (n = 3 independent crosses). Values are mean ± s.e.m. *$p < 0.05$; **$p < 0.01$ (Welch's t test). Source data are provided as a Source Data file.

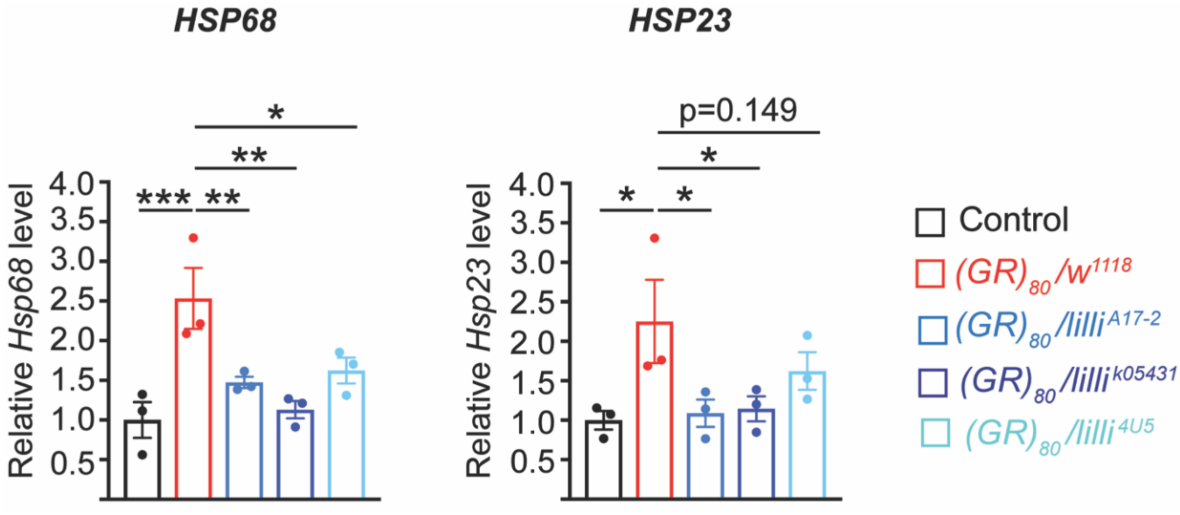

Supplementary Figure 9. Partial loss of Lilli activity decreases $(GR)_{80}$-induced upregulation of *Hsp68* and *Hsp23*. **a** QPCR analysis of *Hsp68* mRNA level in 2-week-old fly heads. **b** QPCR analysis of *Hsp23* mRNA level in 2-week-old fly heads. Each data point is from an independent cross (n = 3 independent crosses). Total RNA from 30–50 flies from each cross was analyzed. Values are mean ± s.e.m. $*p < 0.05$, $**p < 0.01$, $***p < 0.001$ (one-way ANOVA, Dunnett's multiple comparison test). Source data are provided as a Source Data file.

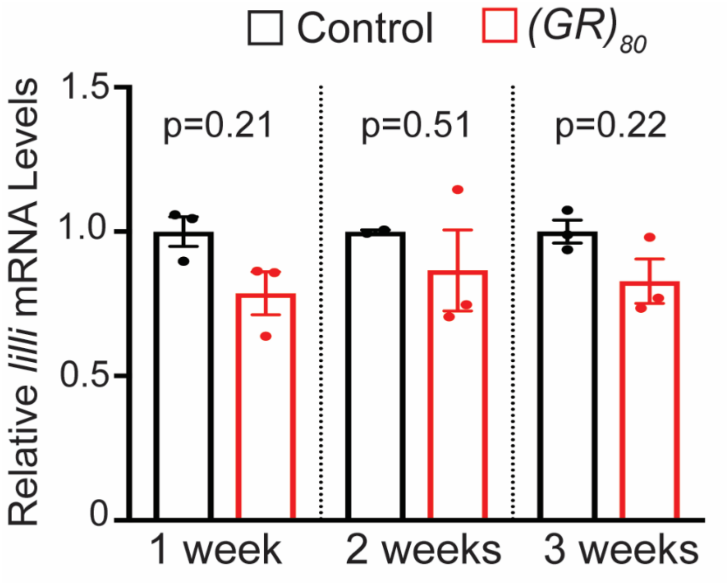

Supplementary Figure 10. $(GR)_{80}$ expression did not affect *lilli* mRNA levels. QPCR analysis of *lilli* mRNA levels in 1–3-week-old fly heads. Each data point is from an independent cross (n = 3 independent crosses). Values are mean ± s.e.m. (multiple *t* test, corrected for multiple comparisons using Holm-Sidak method). Source data are provided as a Source Data file.

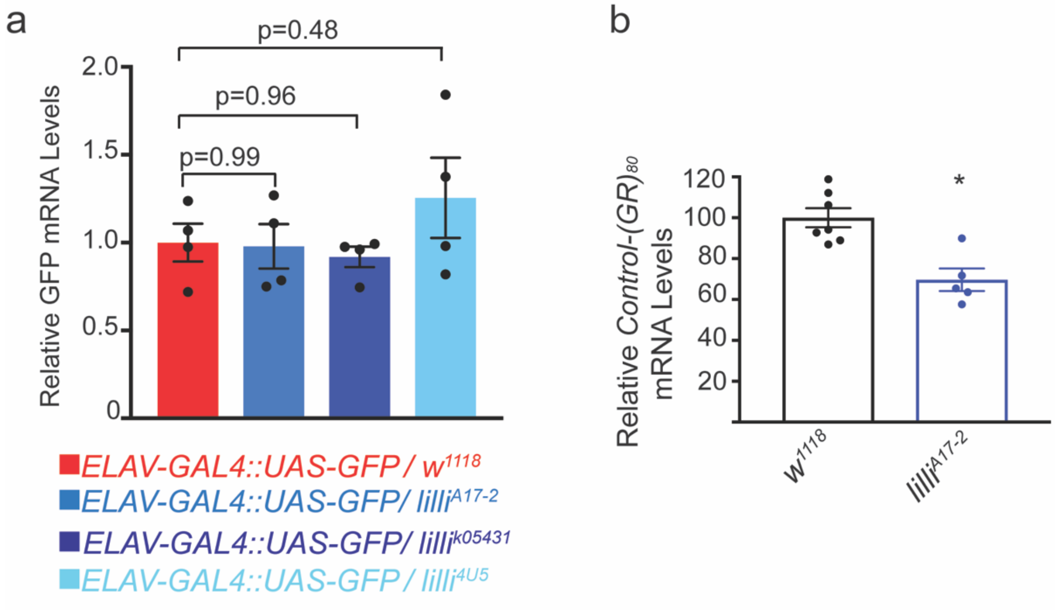

Supplementary Figure 11. Lilli specifically modulates the level of GC rich RNAs. **a** Four independent qPCR measurements of *GFP* mRNA levels in heads of flies from two independent crosses (n = 4 independent crosses). Reducing or increasing Lilli activity did not change *GFP* mRNA levels. Values are mean ± s.e.m. (one-way ANOVA, Dunnett's multiple comparison test). **b** QPCR analysis of (*CONT-GR*)$_{80}$ mRNA levels in the fly heads with or without reduced *lilli* activity. Each data point is from an independent cross (n= 7, 5 independent crosses for WT or *lilli* mutant flies, respectively). Values are mean ± s.e.m. **p* < 0.05 (Mann-Whitney test). Source data are provided as a Source Data file.

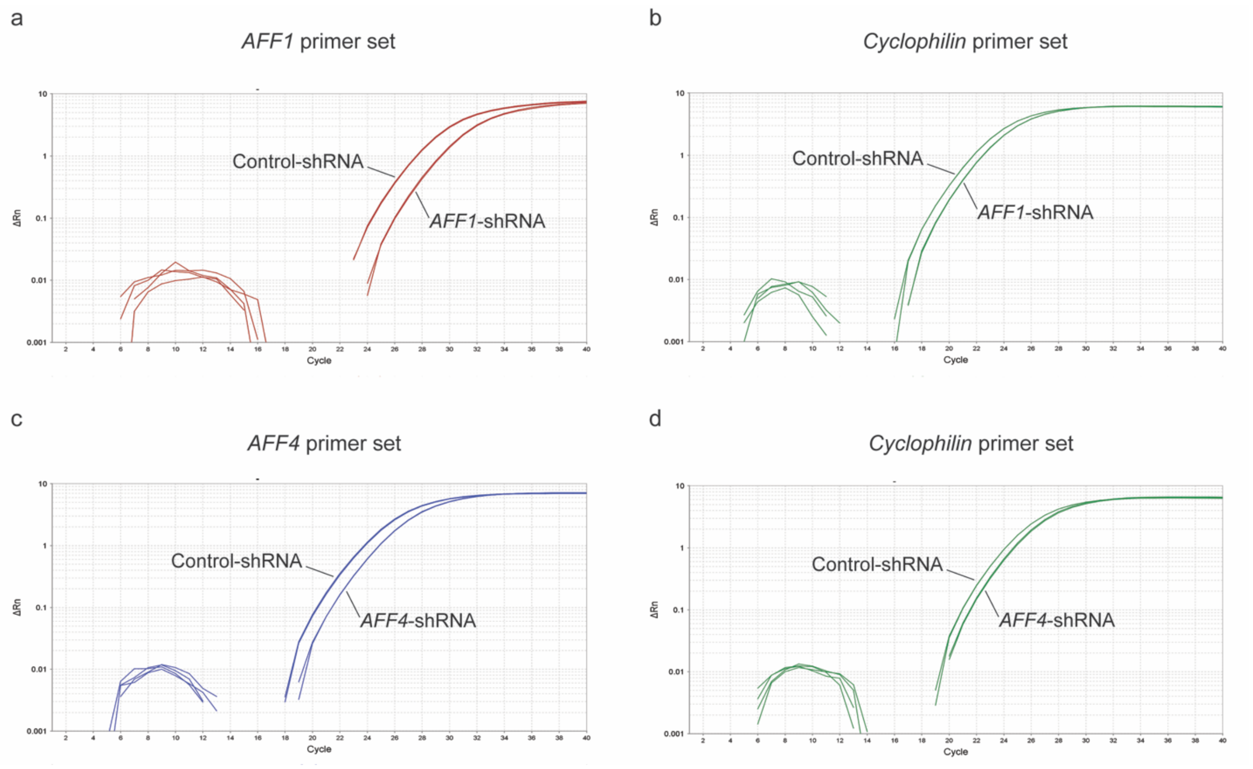

Supplementary Figure 12. Knockdown of *AFF1* or *AFF4* affects control reference genes. **a,b** QPCR amplification curves for *AFF1* and cyclophilin in neurons transduced with control shRNA or *AFF1* shRNA. **c**,**d** QPCR amplification curves for *AFF4* and cyclophilin in neurons transduced with control shRNA or *AFF4* shRNA. The increase in cycle number after *AFF1* or *AFF4* shRNA transduction indicates a decrease in mRNA expression of both *AFF1* or *AFF4* and cyclophilin, the reference control gene. The similar result was obtained for other reference control genes, including *ACTN*, *GAPDH*, *HPRT,* and *SUPT5H*.

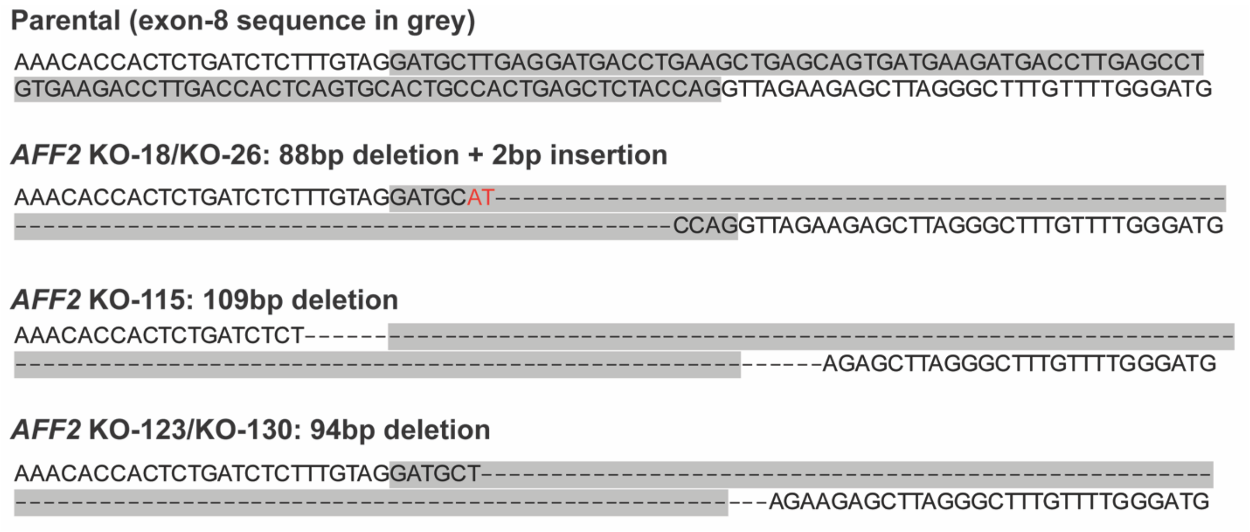

Supplementary Figure 13. Sequencing data from each of the *AFF2*-KO iPSC lines generated compared to the parental line.

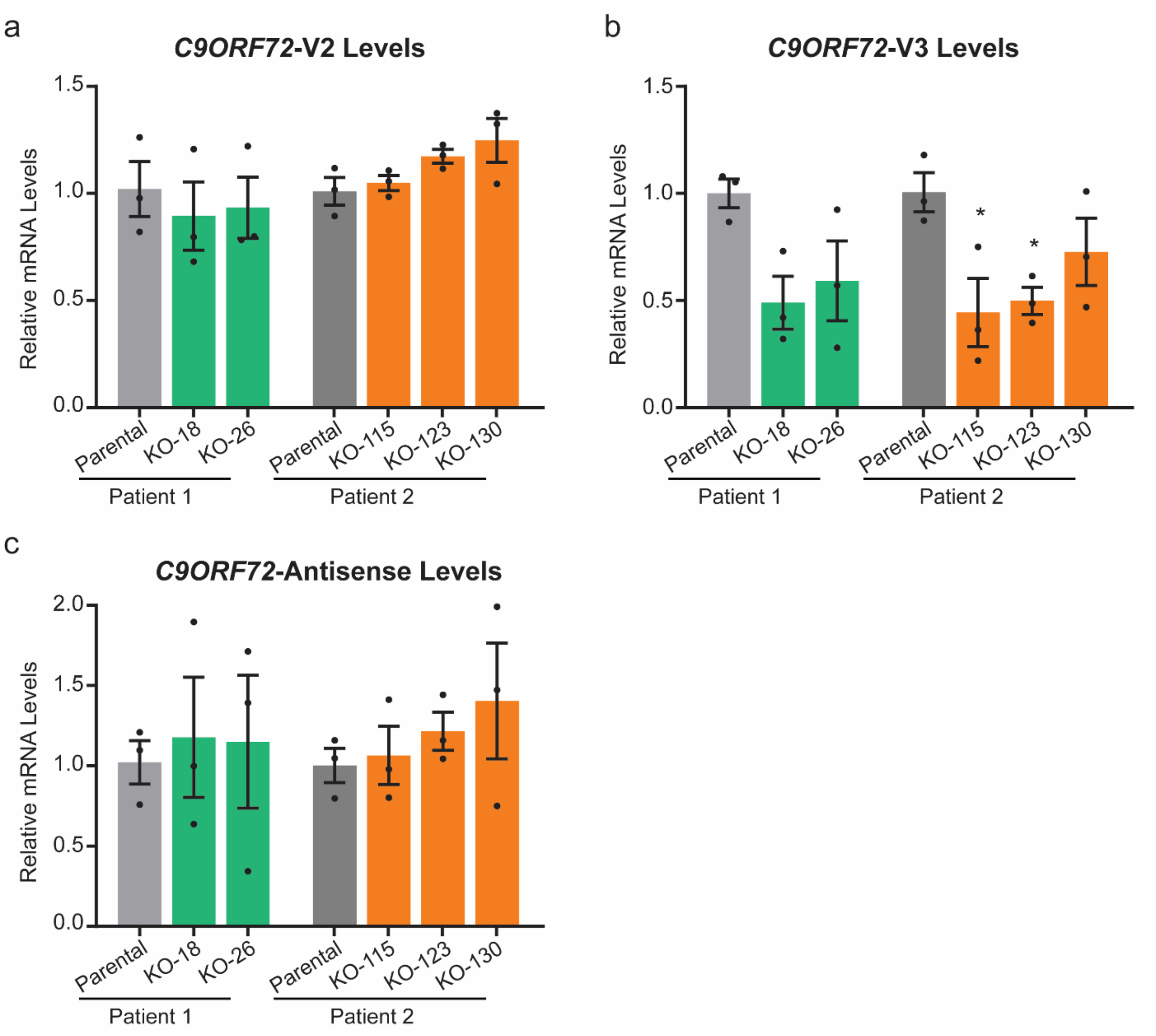

Supplementary Figure 14. mRNA levels of *C9ORF72*-V2 (**a**), *C9ORF72*-V3 (**b**), and *C9ORF72*-antisense (**c**) in *AFF2*-KO iPSC lines. Values are mean ± s.e.m of data from three independent cultures. *$p < 0.05$ (one-way ANOVA, Dunnett's multiple comparisons test)**.** Source data are provided as a Source Data file.

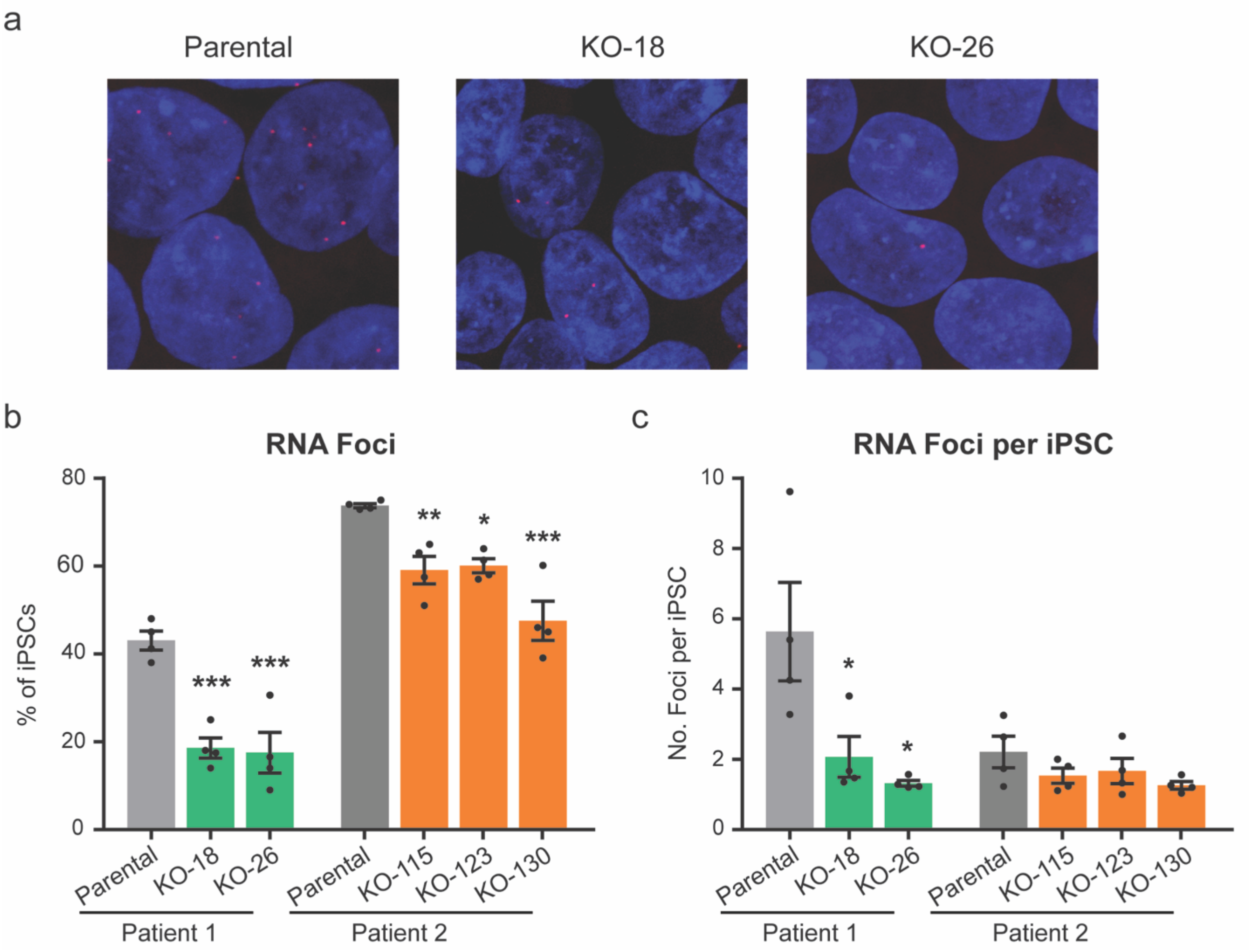

Supplementary Figure 15. a Representative images of the fluorescence *in situ* hybridization (FISH) analysis of parental and respective *AFF2*-KO lines with a Cy3-conjugated $(G_2C_4)_4$ probe. RNA foci are red and the nucleus is blue. **b** Percentage of iPSCs containing RNA foci labelled with a Cy3-conjugated $(G_2C_4)_4$ probe. **c** Average number of RNA foci per cell in *AFF2*-KO iPSC lines. Values are mean ± s.e.m. of data from four independent cultures. $*p < 0.05$; $**p < 0.01$; $***p < 0.001$ (one-way ANOVA, Dunnett's multiple comparisons test). Source data are provided as a Source Data file.

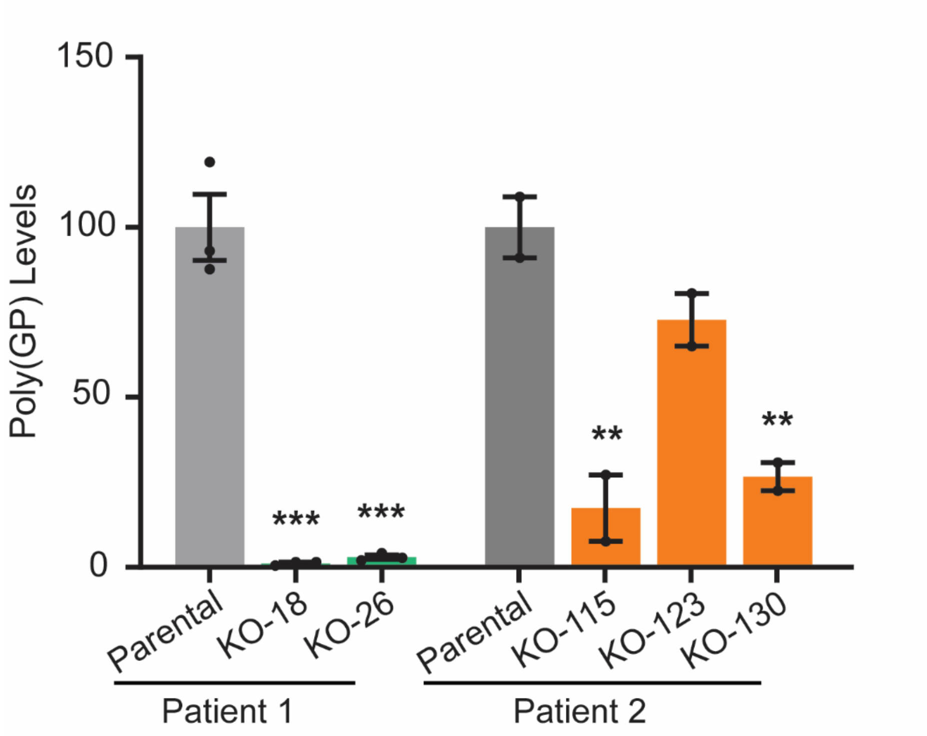

Supplementary Figure 16. Poly(GP) levels in *AFF2*-KO iPSCs measured by immunoassay. Values are mean ± s.e.m. of data from three (patient 1) and two (patient 2) independent cultures. $^{**}p < 0.01$; $^{***}p < 0.001$ (one-way ANOVA, Dunnett's multiple comparisons test). Source data are provided as a Source Data file.

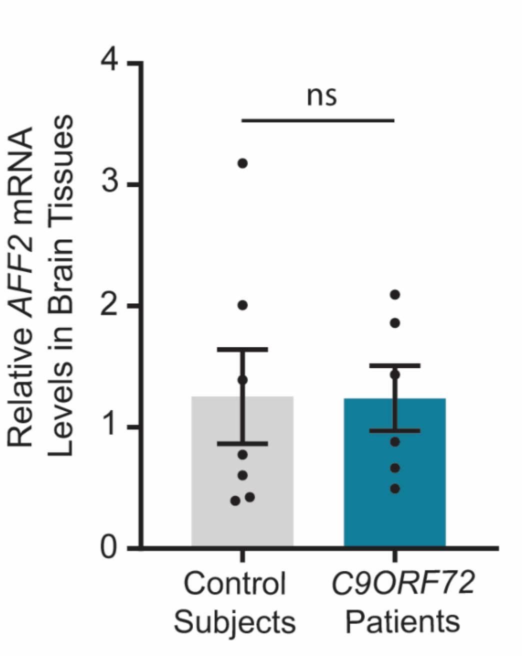

Supplementary Figure 17. Quantification of the *AFF2* mRNA levels in seven control subjects and six *C9ORF72* patients. Values are mean ± s.e.m. ns, not significant. Source data are provided as a Source Data file.

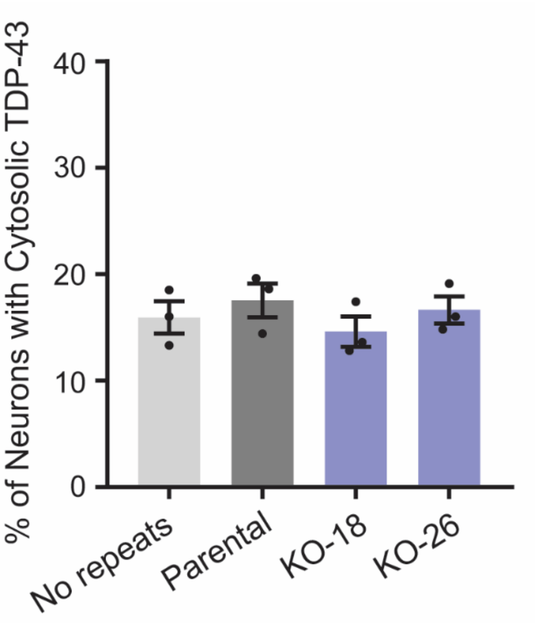

Supplementary Figure 18. Quantification of the percentage of *C9ORF72* iPSC-derived neurons showing cytoplasmic TDP-43 when cultures were kept in the presence of all the neurotrophic factors. Values are mean ± s.e.m. of data from three independent differentiations. Source data are provided as a Source Data file.

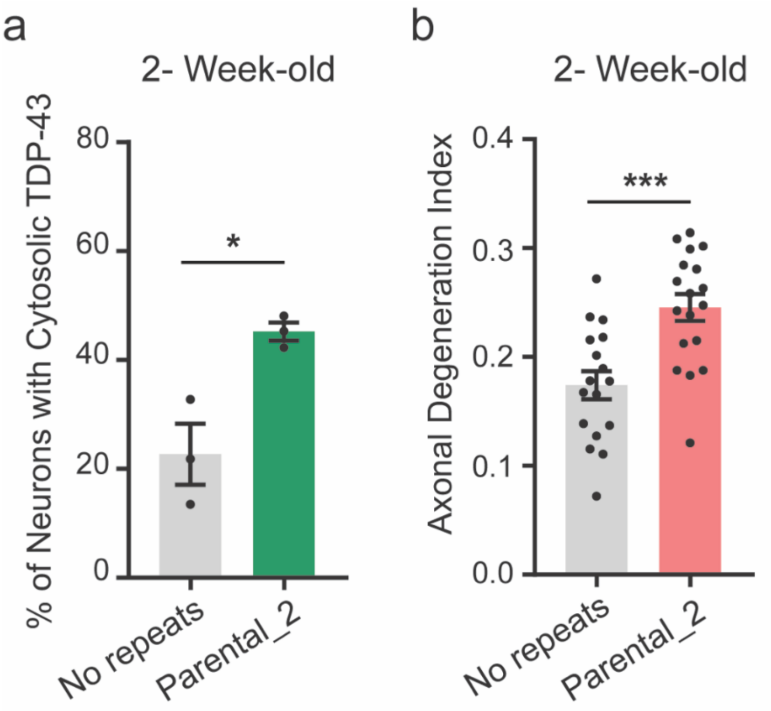

Supplementary Figure 19. TDP-43 and axonal pathology in neurons derived from a second *C9ORF72* patient. **a** Quantification of the percentage of *C9ORF72* iPSC-derived neurons showing cytoplasmic TDP-43 after neurotrophic factor withdrawal for 2-weeks. **b** Quantification of axonal degeneration by measuring the ratio of fragmented axons over the total $TUJ1^+$ area 2-week after neurotrophic factor withdrawal. Six randomly selected fields were analyzed for each condition and per neuronal culture. Each data point represents one field. Data is from 3 independent differentiations. Values are mean ± s.e.m. $*p < 0.05$; $***p < 0.001$ (two-tailed Student's t test). Source data are provided as a Source Data file.

Supplementary Table 1. Primers used in this study.

Primer Name	Primer Sequence
$(GR)_{80}$-F	CTGCAACTACTGAAATCTGCCA
$(GR)_{80}$-R	CGTCGTCGTCCTTGTAATCCA
CONT-$(GR)_{80}$-R	CGTCGTCGTCCTTGTAATCCT
TUB-84F	CACACCACCCTGGAGCATTC
TUB-84R	CCAATCAGACGGTTCAGGTTG
Lilli-F	CGGCCAGTAAGCGACCTG
Lilli-R	AGCAGGTTGTAGTTCTCGCG
C9-1	ACGTAACCTACGGTGTCCC
C9-3	CACTGCATTCCAACTGTCAC
C9-11	CGTCATCGCACATAGAAAACAG
C9-12	AGCGTCATCTTTTACGTGGG
18S-F	TAGACCGAGAGGTCCGGGTA
18S-R	CAAAGGGCAGGGACGTAATCAA
28S-F1	AATGGATGGCGCTTAAGTTG
28S-R1	GTCCTCCAAGGTCTCATTCG
28S-F2	TAAATATGGCGGTCGTGCTC
28S-R2	TTTTCAAGGTCCGAGGAGAA
5.8S-F	CGATGAAGAACGCAGCAAACTG
5.8S-R	CATGGACTGCGATATGCGTTCA
GFP-F	TAAACGGCCACAAGTTCAGC
GFP-R	CTTCATGTGGTCGGGGTAGC
AFF2-PCR-F	TGCCACATCCCACATACACA
AFF2-PCR-R	AGAGATGGGAGGGGGTTGAA
C9ORF72-V2-F	CGGTGGCGAGTGGATATCT
C9ORF72-V2-R	GCCCAAATGTGCCTTACTCT
C9ORF72-V3-F	GGGTCTAGCAAGAGCAGGTG
C9ORF72-V3-R	AGCCCAAATGTGCCTTACTC
AFF2-F	TGTGCAAAGCTGTCCTTTTG
AFF2-R	CTGCCAGTTATGGACCCACT
AFF3-F	CAGCCTCTTCCAGCAAAAAG
AFF3-R	GTTTCTGCCTCTTGCAGTCC
C9ORF72-AS-RT-R	CGACTGGAGCACGAGGACACTGACGAGTGGGTGAGTGAGGAG
C9ORF72-AS-F	AGTCGCTAGAGGCGAAAGC
C9ORF72-AS-R	CGACTGGAGCACGAGGACACTGA
Cyclophilin-F	TGCCATCGCCAAGGAGTAG
Cyclophilin-R	TGCACAGACGGTCACTCAAA

References:

1. Ahmad, S. T., Sweeney, S. T., Lee, J.-A., Sweeney, N. T. & Gao, F.-B. Genetic screen identifies serpin5 as a regulator of the toll pathway and CHMP2B toxicity associated with frontotemporal dementia. *Proc. Natl. Acad. Sci. U. S. A.* **106**, 12168–73 (2009).
