# Supplementary material for: Transcription elongation factor AFF2/FMR2 regulates expression of expanded GGGGCC repeat-containing C9ORF72 allele in ALS/FTD
Source: Nat Commun. 2019 Nov 29;10:5466. doi: 10.1038/s41467-019-13477-8 (PMC6884579; doi:10.1038/s41467-019-13477-8)
Supplement: Supplementary file 3 — Reporting Summary [file 41467_2019_13477_MOESM3_ESM.pdf]

nature research

Corresponding author(s): Fen-Biao Gao

Last updated by author(s): Oct 17, 2019

# Reporting Summary

Nature Research wishes to improve the reproducibility of the work that we publish. This form provides structure for consistency and transparency in reporting. For further information on Nature Research policies, see Authors & Referees and the Editorial Policy Checklist.

## Statistics

For all statistical analyses, confirm that the following items are present in the figure legend, table legend, main text, or Methods section.

| n/a | Confirmed | |
|---|---|---|
| ☐ | ☒ | The exact sample size (*n*) for each experimental group/condition, given as a discrete number and unit of measurement |
| ☐ | ☒ | A statement on whether measurements were taken from distinct samples or whether the same sample was measured repeatedly |
| ☐ | ☒ | The statistical test(s) used AND whether they are one- or two-sided<br>*Only common tests should be described solely by name; describe more complex techniques in the Methods section.* |
| ☒ | ☐ | A description of all covariates tested |
| ☐ | ☒ | A description of any assumptions or corrections, such as tests of normality and adjustment for multiple comparisons |
| ☐ | ☒ | A full description of the statistical parameters including central tendency (e.g. means) or other basic estimates (e.g. regression coefficient) AND variation (e.g. standard deviation) or associated estimates of uncertainty (e.g. confidence intervals) |
| ☐ | ☒ | For null hypothesis testing, the test statistic (e.g. *F*, *t*, *r*) with confidence intervals, effect sizes, degrees of freedom and *P* value noted<br>*Give P values as exact values whenever suitable.* |
| ☒ | ☐ | For Bayesian analysis, information on the choice of priors and Markov chain Monte Carlo settings |
| ☒ | ☐ | For hierarchical and complex designs, identification of the appropriate level for tests and full reporting of outcomes |
| ☒ | ☐ | Estimates of effect sizes (e.g. Cohen's *d*, Pearson's *r*), indicating how they were calculated |

*Our web collection on statistics for biologists contains articles on many of the points above.*

## Software and code

Policy information about availability of computer code

| | |
|---|---|
| Data collection | ImageJ was used for immunostaining analysis. |
| Data analysis | Statistical analyzes were performed using GraphPad Prism (Graphpad Software v.7.04). |

For manuscripts utilizing custom algorithms or software that are central to the research but not yet described in published literature, software must be made available to editors/reviewers. We strongly encourage code deposition in a community repository (e.g. GitHub). See the Nature Research guidelines for submitting code & software for further information.

## Data

Policy information about availability of data

All manuscripts must include a data availability statement. This statement should provide the following information, where applicable:

- Accession codes, unique identifiers, or web links for publicly available datasets
- A list of figures that have associated raw data
- A description of any restrictions on data availability

The data that supports the findings of this study are available from the corresponding authors upon request.

# Field-specific reporting

Please select the one below that is the best fit for your research. If you are not sure, read the appropriate sections before making your selection.

☒ Life sciences ☐ Behavioural & social sciences ☐ Ecological, evolutionary & environmental sciences

For a reference copy of the document with all sections, see nature.com/documents/nr-reporting-summary-flat.pdf

# Life sciences study design

All studies must disclose on these points even when the disclosure is negative.

| | |
|---|---|
| Sample size | All data resulted from at least 3 independent differentiations or iPSCs cultures consisting out of at least 2 technical replicates. For Drosophila experiments, results were gathered from at least 3 independent crosses. |
| Data exclusions | No data exclusion. |
| Replication | All attempts at replication were successfully confirming the findings presented in this study. |
| Randomization | Randomization is not applicable to the current study. Flies and cells were grouped based on the genotype and compared with the respective control. |
| Blinding | The experiments were not performed with blinding. |

# Reporting for specific materials, systems and methods

We require information from authors about some types of materials, experimental systems and methods used in many studies. Here, indicate whether each material, system or method listed is relevant to your study. If you are not sure if a list item applies to your research, read the appropriate section before selecting a response.

## Materials & experimental systems

| n/a | Involved in the study | |
|---|---|---|
| ☐ | ☒ | Antibodies |
| ☐ | ☒ | Eukaryotic cell lines |
| ☒ | ☐ | Palaeontology |
| ☐ | ☒ | Animals and other organisms |
| ☒ | ☐ | Human research participants |
| ☒ | ☐ | Clinical data |

## Methods

| n/a | Involved in the study | |
|---|---|---|
| ☒ | ☐ | ChIP-seq |
| ☒ | ☐ | Flow cytometry |
| ☒ | ☐ | MRI-based neuroimaging |

## Antibodies

| | |
|---|---|
| Antibodies used | The information for all the antibodies used in this study is provided in the material and methods section. |
| Validation | Validation data are shown for each antibody by the manufacturers. For the TBPH antibody validation is shown in Supplementary Figure 4. |

## Eukaryotic cell lines

Policy information about cell lines

| | |
|---|---|
| Cell line source(s) | Patient 1 parental iPSC line was from Almeida et al, Acta Neuropathol, 2013 and patient 2 parental iPSC line was from Sareen et al, Sci Transl Med, 2013. Isogenic no repeat iPSC lines were from Lopez-Gonzalez et al, PNAS, 2019. |
| Authentication | The deletion introduced in the KO iPSCs was confirmed by sequencing of the genomic DNA. |
| Mycoplasma contamination | Cultures were routinely analyzed for mycoplasma contamination using MycoAlert Mycoplasma Detection Kit (Lonza, Catalog #LT07-218). |
| Commonly misidentified lines (See ICLAC register) | Not applicable. |

## Animals and other organisms

Policy information about studies involving animals; ARRIVE guidelines recommended for reporting animal research

| | |
|---|---|
| Laboratory animals | Drosophila melanogaster were used at different aged as stated in the manuscript. |
| Wild animals | Not applicable. |
| Field-collected samples | Not applicable. |
| Ethics oversight | Not applicable. |

Note that full information on the approval of the study protocol must also be provided in the manuscript.
